# Estrogen receptor α in T cells suppresses follicular helper T cell responses and prevents autoimmunity

**DOI:** 10.1038/s12276-019-0237-z

**Published:** 2019-04-15

**Authors:** Do-Hyun Kim, Hong-Jai Park, Hyeon-Soo Park, Jae-Ung Lee, CheMyong Ko, Myung Chan Gye, Je-Min Choi

**Affiliations:** 1 Department of Life Science, College of Natural Sciences, Hanyang University, Seoul, 04763 Republic of Korea; 2 Research Institute for Natural Sciences, Hanyang University, Seoul, 04763 Republic of Korea; 3 Department of Internal Medicine, Yale University School of Medicine, New Haven, CT 06520 USA; 4 Department of Comparative Biosciences, College of Veterinary Medicine, University of Illinois at Urbana Champaign, Urbana, IL 61802 USA

**Keywords:** Autoimmunity, Follicular T-helper cells

## Abstract

Estrogen receptor alpha (ERα) is a sex hormone nuclear receptor that regulates various physiological events, including the immune response. Although there have been some recent studies on ERα regarding subsets of T cells, such as Th1, Th2, Th17, and Treg cells, its role in follicular helper T (TFH) cells has not yet been elucidated. To determine whether ERα controls TFH response and antibody production, we generated T cell-specific ERα knockout (KO) mice by utilizing the CD4-Cre/ERα flox system (CD4-ERα KO) and then analyzed their phenotype. At approximately 1 year of age, CD4-ERα KO mice spontaneously showed mild autoimmunity with increased autoantibody production and CD4^+^CD44^+^CXCR5^+^Bcl-6^+^ TFH cells in the mesenteric lymph nodes and spleen. We next immunized 6–8-week-old CD4-ERα KO mice with sheep red blood cells (SRBCs), which resulted in an increased proportion of TFH cells and germinal center (GC) responses. In addition, 17β-estradiol (E2) treatment decreased TFH responses in wild-type mice and suppressed the mRNA expression of Bcl-6 and IL-21. Finally, we confirmed that the production of high-affinity antigen-specific antibodies and isotype class switching induced by NP-conjugated ovalbumin immunization were elevated in CD4-ERα KO mice under sufficient estrogen conditions. These results collectively demonstrate that the female sex hormone receptor ERα inhibits the TFH cell response and GC reaction to control autoantibody production, which was related to estrogen signaling and autoimmunity.

## Autoimmune disease: the importance of estrogen

Estrogen signaling may play a crucial role in mediating autoimmune responses and preventing diseases such as rheumatoid arthritis. Estrogen plays vital roles in the female endocrine and reproductive systems, and its function is partly mediated by the estrogen receptor alpha (ERα). ERα-deficient mice have more severe autoimmune disease, suggesting a link between estrogen signaling and immune responses. Je-Min Choi at Hanyang University in Seoul, South Korea, and co-workers generated mice with T cells lacking ERα and followed their immune system development. They were particularly interested in monitoring follicular helper T cells (TFH), which help B cells generate antibodies in response to pathogen attack as well as self-antigen. The ERα-deficiency in T cells spontaneously developed mild autoimmune symptoms in mice, with increased levels of TFH and autoantibodies. Their results suggest ERα-mediated estrogen signaling in CD4 T cells is needed to suppress TFH activity and limit autoimmune disease development in female.

## Introduction

Estrogen is the predominant hormone in females and plays important roles in the endocrine and reproductive systems^1^. The function of estrogen is mediated by ERα and ERβ, and both receptors are expressed in most tissues^2^. Although their principle role has been associated with physiological events, such as the menstrual cycle and menopause, previous studies have shown that ER signaling is also involved in the regulation of immune cell functions^3–5^. The role of ERα has been studied in effector T cells, including Th1, Th2, Th17, and Treg cells. ERα has been reported to increase Th2 and Treg cells in mice by interacting with transcription factors, such as GATA3 and Foxp3^6–8^. Recently, ERα has been shown to directly bind to the promoter region of RORγt to suppress Th17 differentiation and function^9,10^. Furthermore, estradiol treatment prevented experimental autoimmune encephalomyelitis (EAE) disease progression by inhibiting the infiltration of Th1 and Th17 cells, while mice with ERα-deficient T cells failed to suppress the disease pathogenesis^11^. These previous studies revealed significant roles of estrogen and estrogen receptors in T cell immunity and autoimmune disease.

Previous studies have suggested that TFH cells stimulate autoantibody production in germinal centers (GCs), which leads to the development of autoimmune disease^12–15^. Spontaneous GC formation and autoantibody production was observed in experimental SLE models, such as NZB and MRL/lpr mice^16,17^. Sanroque mice showed autoimmune lupus symptoms with an excessive TFH cell count and spontaneous GC formation^18^. IL-21, which is an important cytokine for TFH differentiation and function, was increased in patients with SLE compared with healthy controls^19^, and circulating TFH cells, which have been previously shown to be related to disease severity, were increased in patients with SLE^20^. Therefore, TFH cell functions that stimulate autoantibody production may be related to the onset or lead to the development of autoimmune disease, and thus, the regulatory mechanism of the TFH response should be studied to further understand autoimmunity.

Most autoimmune diseases predominantly occur in women because estrogen signaling contributes to sex-dependent immunity, which regulates T cell functions and autoimmune disease^21–23^. Previous ERα-deficient mouse studies have reported increased severity of autoimmune diseases, such as systemic lupus erythematosus (SLE), rheumatoid arthritis (RA), and EAE^11,24–26^. Here, we hypothesized the possible regulatory role of ERα in TFH cell function and autoantibody response, which could be related to autoimmune disease. We analyzed CD4-ERα knockout (KO) mice, which spontaneously developed mild autoimmune phenotypes with increased autoantibodies and TFH cells. We further confirmed that ERα-mediated estrogen signaling suppressed TFH and GC B cell formation, which leads to the production of high-affinity antibodies and isotype-class switching. Our study reveals roles of ERα in T cells regarding TFH responses, which could explain the importance of estrogen signaling in relation to autoimmunity.

## Materials and methods

### Mice

Esr1^TM1.2MMA^ (ERα^fl/fl^) mice were provided by Dr. CheMyong Ko at the University of Illinois at Urbana-Champaign and Dr. Myung Chan Gye at Hanyang University. ERα^fl/fl^ mice were crossed with CD4-Cre^+/-^ transgenic mice to generate CD4-specific ERα-knockout mice (CD4-ERα KO). As a control group, C57BL/6 mice were purchased from Orient Bio (Daejeon, South Korea), and both mouse strains were maintained at the Hanyang University mouse facilities under pathogen-free conditions with ad libitum feeding. All animal protocols in the present study were approved by the Animal Experimentation Ethics Committee of Hanyang University, and experiments were performed according to the guidelines of the Institutional Animal Care and Use Committee (IACUC) of Hanyang University.

### Flow cytometry

Splenocytes, mesenteric lymph node cells, and inguinal lymph node cells were isolated from the indicated mouse and then stained with anti-mouse CD4-PE/Cy7, CD8-Percp/Cy5.5, CD25-PE, CD69-FITC, CD44-APC-Cy7, CD62L-FITC, B220-Percp/Cy5.5, GL-7-FITC, and CD95-PE antibodies (eBioscience, San Diego, CA, USA) for 15 min at 4 °C. For TFH cell analysis, cells were stained with anti-mouse CXCR5-biotin for 30 min at 4 °C followed by anti-mouse CD44-APC/Cy7 and CD4-PE/Cy7 and streptavidin-APC staining. After fixation and permeabilization using a Foxp3 Staining Kit (eBioscience), intracellular Bcl-6 was stained using anti-mouse Bcl-6-PE (BD Bioscience, San Jose, CA, USA) for 40 min at room temperature. Cells were examined using a FACSCanto II system (BD Bioscience), and data were analyzed using FlowJo software (Treestar, Ashland, OR, USA).

### SRBC and NP-OVA immunization

Mice were intraperitoneally (i.p.) immunized with 1 × 10^9^ sheep red blood cells (SRBCs) (Innovative Research, Novi, MN, USA) or subcutaneously with 50 μg of NP-OVA (Bioresearch Technologies, Novato, CA, USA) diluted in Complete Fruend’s Adjuvant (Chondrex, Redmond, WA, USA). Seven days after immunization, the mice were sacrificed, and the splenocytes and inguinal lymph node cells were isolated and analyzed via flow cytometry. The hormone 17β-estradiol (E2) was purchased from Sigma and dissolved in DMSO. To assess the regulatory effect of E2 on TFH cell differentiation in vivo, 3 mg/kg or 10 mg/kg E2 was injected i.p. following the indicated experimental scheme. Cells from the inguinal lymph node were isolated from the mice for further analysis.

### Immunofluorescence microscopy

For GC formation analysis, the spleens from 1-year-old mice or SRBC-immunized mice were detached and frozen in OCT compound. Tissues were sectioned to 7 μm thickness, fixed in acetone at −20 °C, washed with PBS, and blocked with 0.1% BSA in PBS for 30 min at room temperature. Tissues were stained with anti-PNA-FITC (Sigma-Aldrich, St. Louis, MO, USA), IgD-PE, and CD4-APC (eBioscience) antibodies diluted in blocking solution overnight at 4 °C. The tissues were incubated with ProLong Gold anti-fade reagent (Life Technologies, Carlsbad, CA, USA) for 30 min at 4 °C, and images were obtained using a Leica DM IRE2 confocal microscope.

### ANA staining

Sera from 1-year-old or 6–8-week-old mice were isolated and stained using an ANAFAST Indirect Fluorescent Antibody kit (Diasorin, Stillwater, MN, USA) following the manufacturer’s instructions.

### In vitro TFH-like cell skewing

CD4^+^CD25^−^CD44^−^CD62L^+^ naïve T cells were isolated from the spleen and inguinal lymph node using a FACS Aria III cell sorter (BD Bioscience). The cells were stimulated by plate-bound anti-CD3 and anti-CD28 antibodies (eBioscience) under cytokine conditions: IL-6 (20 ng/ml), IL-21 (25 ng/ml) (Peprotech, Rocky Hill, NJ, USA), and IL-2 neutralizing antibody (5 μg/ml) (BD bioscience) in RPMI-based cell medium. After 72 h, the cells were collected, and mRNA expression was measured using real-time PCR.

### In vitro T cell differentiation

CD4^+^CD25^−^CD44^−^CD62L^+^ naïve T cells were isolated using a FACS Aria III cell sorter (BD Bioscience). Naïve CD4 T cells were stimulated with 2 μg/mL plate-bound anti-CD3 and anti-CD28 antibodies (eBioscience) under cytokine conditions. Th1: IL-12 (BD bioscience, 0.2 ng/mL), IL-2 (Peprotech, 50 U/mL), and anti-IL-4 antibody (Invitrogen, 5 μg /mL); Th2: IL-4 (Peprotech, 30 ng/mL), IL-2 (Peprotech, 50 U/mL), anti-IFNγ antibody (Invitrogen, 5 μg/mL); Th17: IL-6 (BD bioscience, 30 ng/mL), TGFβ (R&D systems, 0.5 ng/mL), IL-1β (R&D systems, 20 ng/mL), IL-23 (BD bioscience, 20 ng/mL), anti-IFNγ antibody (5 μg/mL), and anti-IL-4 antibody (5 μg/mL); and Treg: TGFβ (5 ng/mL) and IL-2 (100 U/mL) in RPMI-based cell medium. After 3 days (Th1, 17, Treg) or 6 days (Th2), the cells were analyzed via flow cytometry.

### Real-time PCR

RNA was isolated using an RNeasy mini kit (Qiagen, Valencia, CA, USA) according to the manufacturer’s protocol. RNA yield and purity were determined with a NanoDrop spectrophotometer. Total RNA (500 ng) was used for cDNA synthesis in a 20-μL reaction volume using qPCR RT Master Mix (Toyobo, Osaka, Japan). Real-time PCR was performed using iQ SYBR Green Supermix (Bio-Rad, Hercules, CA, USA). Actin was used as the housekeeping gene. The primer sequences used for the experiment are listed in Supplementary Table 1.

### Enzyme-linked immunosorbent assay (ELISA)

Antibody production in mouse serum was measured by using mouse anti-dsDNA IgG ELISA kit (Chondrex); IgM ELISA kit (eBioscience); IgG ELISA kit (Abcam, Cambridge, England); HRP-conjugated goat anti-mouse IgG1, IgG2b, IgG2c, and IgG3 antibody (Southern Biotech, Birmingham, AL, USA) according to the manufacturer’s instructions. In some experiment, NP-conjugated BSA (Biosearch Technologies, Petaluma, CA, USA) was coated for measuring NP-specific antibody production.

### Statistical analysis

Data were analyzed using Student’s t-test, and one-way ANOVA was used for multiple comparisons using Prism5 software (GraphPad, San Diego, CA, USA). P-values less than 0.05 were considered statistically significant.

## Results

### ERα deficiency in T cells spontaneously produced autoantibodies in aged mice

**Fig. 1:**
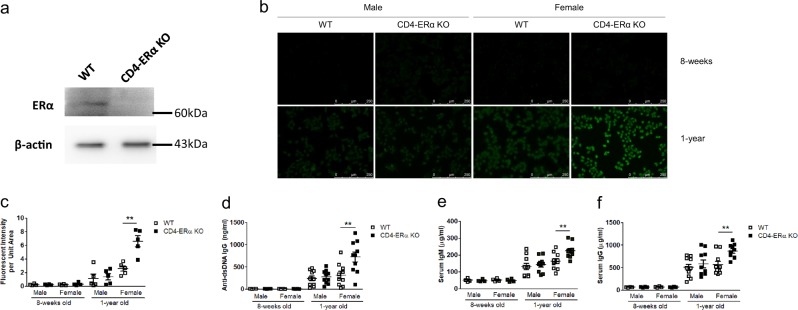
One-year-old CD4-ERα knockout female mice developed spontaneous mild autoimmune phenotypes. **a** Naïve CD4 T cells were isolated from the spleen of WT and generated CD4-ERα KO mice, and the ERα protein expression level in CD4 T cells was confirmed by western blotting. **b, c** The amounts of anti-nuclear antibody in the serum of 1-year-old mice were examined via immunofluorescence of prefixed Hep-2 cells stained with diluted (1:40) sera followed by Alexa Fluor 488-conjugated goat anti-mouse Ig antibody. **d**–**f** ELISAs were performed for quantitation of (**d**) anti-dsDNA, (**e**) IgM and (**f**) total IgG in the sera of 1-year-old mice. The values represent the mean ± SEM; *n* = 5. **P < 0.01

To determine the role of ERα in TFH functions and antibody production, we generated mice lacking ERα in T cells (CD4-ERα KO) by using the CD4-Cre/ERα flox system. Naïve T cells from CD4-ERα KO mice showed non-detectable ERα levels compared with wild-type controls (Fig. 1a). There was no significant difference in the immune cell population in 6–8-week-old male and female mice (Supplementary Fig. 1). However, when we examined the levels of autoantibodies in serum from 1-year-old CD4-ERα KO mice, we found higher anti-nuclear antibody (ANA) levels in CD4-ERα KO female mice than in the wild-type littermate control group, while 8-week-old mice did not show any differences (Fig. 1b, c). The anti-dsDNA antibody production was also significantly increased in 1-year-old CD4-ERα KO female mice compared with that in the control group (Fig. 1d). In addition, total serum IgG and IgM levels were also significantly increased in CD4-ERα KO female mice compared with control mice (Fig. 1e, f). Due to the increased autoantibody levels in CD4-ERα KO mice, we hypothesized that there could be increased T cell activation in the mice. However, there were no significant differences in activation markers on CD4 T cells, such as CD69, CD25, and CD44, in the spleen, mesenteric lymph nodes (MLNs), and inguinal lymph nodes (ILNs) from 1-year-old mice (Supplementary Fig. 2). These results suggest that ERα in T cells can inhibit spontaneous production of autoantibodies without interfering with T cell activation.

### ERα deficiency in T cells spontaneously increased TFH cells and GC B cells in aged mice

**Fig. 2:**
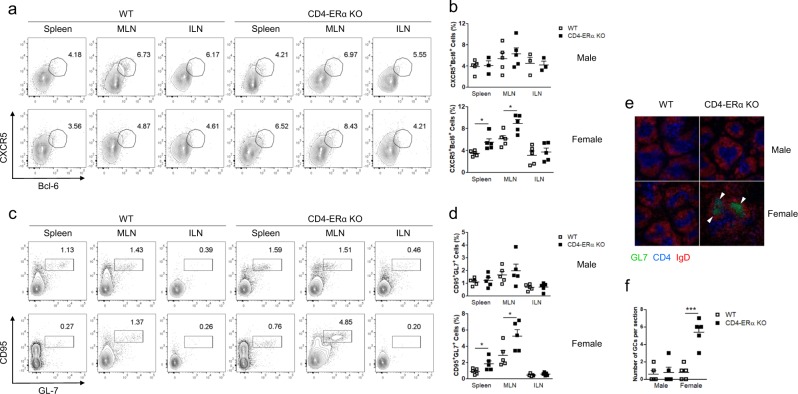
One-year-old female CD4-ERα knockout (KO) mice spontaneously induced follicular helper T (TFH) and germinal center (GC) B cells. **a**–**d** Lymphocytes from the spleen, mesenteric lymph nodes (MLNs), and inguinal lymph nodes (ILNs) were isolated from 1-year-old wild-type (WT) and CD4-ERα KO mice, and then, the cells were stained with anti-CD4, anti-CD44, anti-CXCR5, anti-Bcl-6, anti-B220, anti-CD95, and anti-GL-7 antibodies for flow cytometric analysis. **a**, **b** CD4^+^CD44^+^CXCR5^+^Bcl-6^+^ TFH cells and **c**, **d** B220^+^CD95^+^GL-7^+^ GC B cells were analyzed. **e**, **f** Spleen sections from 1-year-old WT and CD4-ERα KO mice were stained for PNA (green), IgD (red), and CD4 (blue) to detect GCs, B cells, and T cells, respectively. Stained slides were visualized using fluorescence microscopy. The values represent the mean ± SEM; *n* = 5. *P < 0.05; and ***P < 0.001

Because we found increased autoantibody levels in 1-year-old CD4-ERα KO female mice without an effect on T cell activation, we hypothesized that the proportion of TFH cells might be increased in lymphoid tissues. Therefore, we examined the proportion of CD4^+^CD44^+^CXCR5^+^Bcl-6^+^ TFH cells and CD95^+^GL-7^+^ GC B cells in the spleen, MLNs, and ILNs of the mice. The gating strategy for the TFH population is indicated in Supplementary Fig. 3, which shows that the Bcl-6^+^CXCR5^+^ cells were from the CD4^+^CD44^high^ population. We found that the proportion of TFH cells in the spleen and MLNs was significantly increased in CD4-ERα KO female mice compared to littermate control mice, while there were no differences in the ILNs (Fig. 2a, b). Furthermore, the proportion of GC B cells was significantly higher in female CD4-ERα KO mice than in wild-type mice, without notable differences in male mice (Fig. 2c, d). Immunostaining of spleen sections revealed that the number of GCs was significantly increased in CD4-ERα KO female mice compared with wild-type mice (Fig. 2e, f), suggesting that ERα in T cells possibly regulates spontaneous induction of TFH responses and autoantibody production to prevent autoimmunity.

### ERα deficiency in T cells increased TFH cell and GC responses following SRBC immunization

**Fig. 3:**
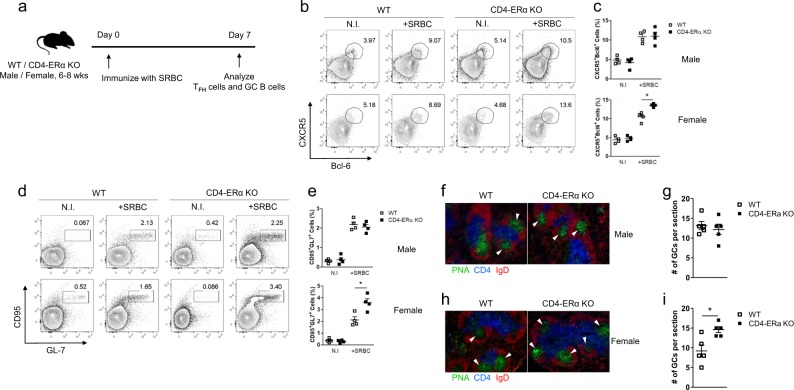
Follicular helper T (TFH) and germinal center (GC) responses were increased in young female CD4-ERα knockout (KO) mice by sheep red blood cell (SRBC) immunization. **a** Experimental scheme of SRBC immunization. **b**–**e** Flow cytometric analysis of (**b**, **c**) CD4^+^CD44^+^CXCR5^+^Bcl-6^+^ TFH cells and **d**, **e** B220^+^CD95^+^GL7^+^ GC B cells in spleens from 6–8-week-old wild-type (WT) and CD4-ERα KO male and female mice with or without SRBC immunization. **f**–**i** Spleen sections from SRBC-immunized WT and CD4-ERα KO mice were stained for PNA (green), IgD (red), and CD4 (blue) to detect germinal centers, B cells, and T cells, respectively. The stained slides were analyzed via fluorescence microscopy. The values represent the mean ± SEM; *n* = 4. *P < 0.05

Aged female mice with T cells lacking ERα showed a spontaneous mild autoimmune phenotype with increased autoantibody production, and thus, we hypothesized that ERα deficiency in T cells increases the TFH response upon antigen challenge in young mice. We intraperitoneally administered 1 × 10^9^ SRBCs to 6–8-week-old wild-type and CD4-ERα KO mice, and then, the proportion of TFH cells and GC B cells was analyzed on day 7 (Fig. 3a). Following SRBC immunization, the proportion of CD4^+^CD44^+^CXCR5^+^Bcl-6^+^ TFH (Fig. 3b, c) and CD95^+^GL-7^+^ GC B cells (Fig. 3d, e) in the spleen was significantly increased in CD4-ERα KO female mice compared with that in control mice, while male mice showed no differences. Additionally, analysis of splenic sections from SRBC immunized mice via immunostaining with PNA, anti-CD4, and anti-IgD antibodies revealed that the number of GCs was significantly increased in CD4-ERα KO female mice compared with wild-type mice, without any differences in male mice (Fig. 3f–i). These results suggest that ERα in T cells suppressed TFH cell formation and GC responses to SRBC antigens in young females.

### E2 treatment suppresses TFH responses and TFH cell-related gene expression

**Fig. 4:**
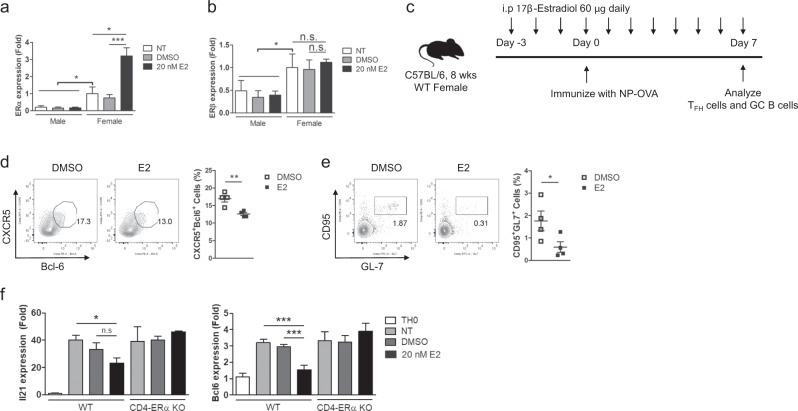
Treatment of wild-type (WT) mice with 17β-estradiol (E2) prevented the induction of follicular helper T (TFH) and germinal center (GC) B cells by NP-OVA immunization. **a**, **b** FACS-sorted naïve CD4 T cells were stimulated by plate-bound anti-CD3 and anti-CD28 antibodies under TFH-like cell skewing conditions for 72 h with or without 20 nM E2. Real-time PCR was performed to measure the mRNA levels of (**a**) ERα and (**b**) ERβ. **c** Experimental scheme of NP (4-hydroxy-3-nitrophenyl acetyl hapten)-conjugated ovalbumin (NP-OVA) immunization and E2 treatment. **d**, **e** Flow cytometric analysis of (**d**) CD4^+^CD44^+^CXCR5^+^Bcl-6^+^ TFH cells and (**e**) B220^+^CD95^+^GL-7^+^ GC B cells from the inguinal lymph nodes (ILNs) of NP-OVA-immunized DMSO control and E2 treated group mice. **f** Naive CD4 + T cells from the spleen and ILNs of 6- to 8-week-old WT and CD4-ERα knockout female mice were isolated and stimulated by plate-bound anti-CD3 and anti-CD28 antibodies under TFH-like cell skewing conditions for 72 h. The indicated target mRNA expression levels were quantified by real-time PCR, and the values were compared between the indicated groups. The values represent the mean ± SEM; *n* = 4. *P < 0.05; and ***P < 0.001

The results showing increased TFH responses and antibody production in both aged and young CD4-ERα KO mice prompted us to investigate the effects of E2 treatment on immunized wild-type mice. We first examined estrogen receptor levels in T cells upon E2 treatment. Basal levels of ERα and ERβ in female T cells were significantly higher than those in male T cells (Fig. 4a, b). When naïve CD4 T cells were stimulated under TFH-like cell skewing conditions with or without 20 nM E2 treatment for 3 days, the mRNA expression level of ERα but not ERβ in E2-treated female T cells was significantly increased compared with that in the control group (Fig. 4a, b), suggesting the possibility of a dominant ERα function in regulating T cell function in females. To examine whether E2 treatment would inhibit TFH formation, we administered 60 μg of E2 daily per mouse, and then, the mice were immunized with NP (4-hydroxy-3-nitrophenyl acetyl hapten)-conjugated ovalbumin (NP-OVA) (Fig. 4c). We found that E2 treatment significantly prevented the induction of TFH and GC B cells in the draining lymph node of immunized mice (Fig. 4d, e). To further confirm these observations, we stimulated and cultured naïve CD4 T cells from wild-type and CD4- ERα KO mice under TFH-like conditions with or without 20 nM E2 for 3 days. We found that the mRNA level of Bcl-6 was significantly decreased in E2-treated T cells, which also showed a reduced pattern of IL-21 expression, but these differences were not apparent in ERα KO T cells (Fig. 4f). Collectively, our data suggest that exogenous E2 treatment in immunized mice prevents TFH responses, and this effect is mediated by ERα signaling related to IL-21 and Bcl-6 expression.

### ERα deficiency in T cells increased high-affinity antigen-specific antibody production and isotype class switching

**Fig. 5:**
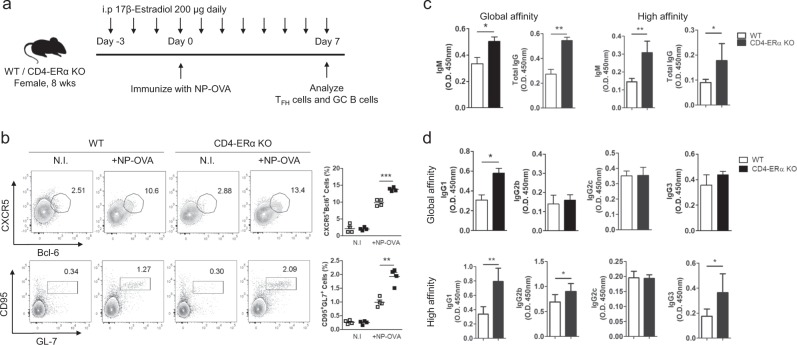
High-affinity antibody production and isotype class switching were increased in female CD4-ERα knockout (KO) mice under an estrogen sufficient environment. **a** Experimental scheme of NP-OVA immunization and 17β-estradiol (E2) treatment. **b** Flow cytometric analysis of CD4^+^CD44^+^CXCR5^+^Bcl-6^+^ follicular helper T (TFH) cells and B220^+^CD95^+^GL-7^+^ germinal center (GC) B cells in inguinal lymph nodes (ILNs) from 8-week-old NP-OVA immunized WT and CD4-ERα KO female mice with 200 µg E2 treatment. **c**, **d** ELISAs for NP_7_-(high affinity) and NP_30_- (low affinity) specific antibodies were performed to measure (**c**) IgM, total IgG, (**d**) IgG1, IgG2b, IgG2c, and IgG3 levels in the sera of NP-OVA-immunized WT and CD4-ERα KO female mice. The values represent the mean ± SEM; *n* = 4. *P < 0.05; **P < 0.01; and ***P < 0.001

Based on our observations of the CD4-ERα KO mice and the E2 treatment study regarding TFH responses, we hypothesized that sufficient estrogen levels in the mice would result in reduced high-affinity antigen-specific antibodies and isotype class switching, which are dependent on ERα. To obtain better resolution, we increased the amount of the daily E2 injection to 200 μg, and mice were immunized with NP-OVA under sufficient estrogen conditions. The TFH and GC B cell proportions were analyzed on day 7 (Fig. 5a). We found increased TFH and GC B cells in CD4-ERα KO mice compared with wild-type mice (Fig. 5b), while male mice showed no significant differences (Supplementary Fig. 4). This result confirmed that the E2-based suppressive effect on the TFH response was dependent on ERα expression in T cells. The proportion of follicular regulatory T (TFR) cells was not altered by E2 treatment in the NP-OVA-immunized mice (Supplementary Fig. 5). Finally, we examined the level of both low- and high-affinity NP-specific antibodies in the sera of NP-OVA-immunized wild-type and CD4-ERα KO mice. There was a significant increase in high- and low-affinity total IgG and IgM (Fig. 5c) and in high-affinity NP-specific IgG1, IgG2b, and IgG3 levels in sera from the NP-OVA-immunized CD4-ERα KO mice compared with wild-type mice (Fig. 5d). Collectively, these results suggest that an estrogen-sufficient environment suppresses TFH functions, such as high-affinity antigen-specific antibody production and isotype class switching, and this effect is dependent on ERα expression in T cells.

## Discussion

Estrogen signaling regulates various biological events through ERα and ERβ. ERα has been studied in effector T cell responses as a suppressor of Th1 and Th17 cells, while it induces Th2 and Treg cells^6,8,10^. Here, we demonstrated the suppressive function of ERα on TFH responses and antibody production in female mice by utilizing T cell-specific ERα KO mice. Exogenous E2 treatment inhibited TFH cell functions and antigen-specific antibody production and reduced IL-21 and Bcl-6 expression. We would like to suggest that reduced estrogen signaling in female mice contributes to abnormal regulation of TFH responses, thereby resulting in the production of autoantibodies.

Although sex hormones mainly control various sex-dependent physiological events, they are also expressed in immune cells to regulate immune responses^3–5^. Androgen/androgen receptor signaling has been shown to stimulate innate immune responses, including neutrophil proliferation and maturation^27^ and TNFα and CCR2 expression in macrophages^28^. Androgen reduces IFNγ production through regulation of STAT4 phosphorylation and PTPN1 expression in CD4 T cells^29,30^. Progesterone has been reported to inhibit TLR-4- and TLR-9-mediated pro-inflammatory cytokine production through suppression of NF-κB p65 activity and induction of SOCS1 mRNA^31^. Furthermore, progesterone can inhibit T cell proliferation and reduce CD25 expression and ZAP-70 phosphorylation^32^, suggesting that the sex hormone progesterone is actively involved in regulation of the immune response.

Estrogen is a female sex hormone that is highly expressed during the late follicular phase of the menstrual cycle^22^. Recent studies have demonstrated that estradiol treatment reduces Th17 differentiation via suppression of IL-23 secretion in dendritic cells^33^, and it also induces the development of tolerogenic dendritic cells and prevents EAE pathogenesis^34^. In addition, E2 treatment resulted in a protective effect on EAE^35^, and another study showed that this effect was dependent on ERα expression in T cells^11^. In the present study, we observed spontaneously increased TFH cell formation and autoantibody production in aged female CD4-ERα KO mice. This mild autoimmune phenotype was not related to reduced regulatory T cells in CD4-ERα KO mice (Supplementary Fig. 6). The increase in TFH cells without ERα among T cells and the reduced Bcl-6 level induced by E2 treatment suggest possible direct ERα regulation of TFH cell-related gene expression. Previously, ERα has been shown to bind to the RORγt promoter region to regulate Th17 differentiation^10^. ERE-like sequences are present in the initial portion (−585~−574, −1229~−1217) of the Bcl-6 promoter, implying there is a possibility of direct control of Bcl-6 expression by ERα (Supplementary Fig. 7). Future studies using chromatin immunoprecipitation assays to investigate the molecular mechanism of Bcl-6 regulation are warranted. In sera from aged CD4-ERα KO mice, increased IFNγ production was detected (Supplementary Fig. 8), suggesting the possibility of spontaneously increased TFH cell formation through increased IFNγ stimulation in vivo. Previously, IFNγ receptor deficiency in sanroque mice resulted in diminished TFH and GC B cells, and STAT1 was suggested to be required for early TFH cell differentiation^36,37^. Interestingly, ERα KO T cells showed significantly increased IFNγ production during Th1 differentiation (Supplementary Fig. 9), suggesting the possibility that increased IFNγ might be related to the increased TFH differentiation in vivo. A previous study by Miyauchi et al. reported that Th1 cells enhance the global antibody response through production of IFN-γ and IL-21^38^. However, TFH cells are an essential T cell subset for affinity maturation and high-affinity antibody production^39^. In the present study, we found a significant increase in high-affinity antibodies in addition to global-affinity antibodies against NP-OVA immunization, suggesting that not only an increase in Th1 cells but also ERα directly regulate TFH responses for antibody production.

It is well known that women have a higher incidence of autoimmune disease than men, which could be related to differential sex hormone-based immune regulation. Previous studies have suggested that the female sex hormone estrogen has protective roles in autoimmune diseases, such as EAE and RA^11,24,40,41^. The relapse rate and inflammatory cytokine production were decreased in pregnant EAE mice with high levels of estrogen; however, this protection was ablated during the postpartum phase^42^. The worsening of disease severity observed in postmenopausal patients with rheumatoid arthritis also suggests a protective role of female sex hormones in autoimmune disease^43^. In the present study, we revealed the role of ERα in TFH responses, which would explain the possible contribution to the progression of autoimmunity in postmenopausal females. In conclusion, our findings suggest that estrogen-ERα signaling suppresses TFH responses and autoantibody production and that a sufficient estrogen level in females would be beneficial to prevent TFH-mediated autoimmunity.

## Supplementary information

Supplementary Figure Legend

Supplementary Table 1

Supplementary Figure 1

Supplementary Figure 2

Supplementary Figure 3

Supplementary Figure 4

Supplementary Figure 5

Supplementary Figure 6

Supplementary Figure 7

Supplementary Figure 8

Supplementary Figure 9
