# Supplementary material for: Estrogen receptor α in T cells suppresses follicular helper T cell responses and prevents autoimmunity
Source: Exp Mol Med. 2019 Apr 15;51(4):41. doi: 10.1038/s12276-019-0237-z (PMC6465332; doi:10.1038/s12276-019-0237-z)
Supplement: Supplementary file 1 — Supplementary Figure Legend [file 12276_2019_237_MOESM1_ESM.docx]

**Supplementary Figure 1. No significant abnormal immunological phenotypes were observed in 6–8-week-old CD4-ERα knockout (KO) mice.**

**a** The proportion of CD4^+^ and CD8^+^ cells of the spleen, inguinal lymph node, mesenteric lymph node, and Peyer’s patch in 8-week-old wild-type and CD4-ERα KO male and female mice was analyzed by flow cytometry. **b** Flow cytometric analysis of the percentage of CD19^+^ and NK1.1^+^ cells of the spleen, inguinal lymph node, mesenteric lymph node, and Peyer’s patch in 8-week-old wild-type and CD4-ERα KO male and female mice.

**Supplementary Figure 2. No significant differences in the expressions of T cell activation markers in 1-year-old CD4-ERα knockout (KO) mice were observed.**

**a-d** Lymphocytes from the spleen, mesenteric lymph nodes (MLN), and inguinal lymph nodes (ILN) were isolated from 1-year-old wild-type and CD4-ERα KO male and female mice, and then immunostained with anti-CD4, anti-CD25, anti-CD69, anti-CD44, anti-CD62L antibodies for flow cytometric analysis. **a, b** CD4^+^CD25^+^ or CD4^+^CD69^+^ activated T cells, and **c, d** CD4^+^CD44^-^CD62L^+^ naive and CD4^+^CD44^+^CD62L^-^ memory CD4 T cells were analyzed. Values represent the mean ± SEM, n=5.

**Supplementary Figure 3. Gating strategy for TFH cell analysis. a** TFH cell gating strategy of representative splenocytes FACS staining from 1-year-old mice. **b** CXCR5^+^Bcl-6^+^ TFH cells from CD4^+^CD44^low^ population of spleen, ILN, MLN from WT, CD4-ERa KO female mice.

**Supplementary Figure 4. No differences were observed for follicular helper T** **(TFH) responses such as antibody production and isotype switching in male mice under estrogen-sufficient conditions.**

The hormone 17β-estradiol (E2) (60 µg) was injected daily to male mice for 3 days prior of NP-OVA immunization, and after immunization, E2 was continuously injected daily for 7 days. The mice were sacrificed on day 7, and the inguinal lymph nodes (ILN) was analyzed by flow cytometry. **a** Flow cytometric analysis of CD4^+^CD44^+^CXCR5^+^Bcl-6^+^ TFH cells in the ILN from 8-week-old NP-conjugated ovalbumin (NP-OVA)-immunized wild-type (WT) and CD4-ERα knockout (KO) male mice with or without the E2 treatment. **b** Bar graph shows the representative data of **a**. c Flow cytometric analysis of B220^+^CD95^+^GL-7^+^ germinal center B cells in the ILN from 8-week-old NP-OVA-immunized WT and CD4-ERα KO male mice with or without the E2 treatment. **d** Bar graph shows the representative data of **c.** **e** low-affinity IgM, total IgG, **d** IgG1, IgG2b, IgG2c, and IgG3 in the sera of NP-OVA-immunized WT and CD4-ERα KO male mice. Values represent the mean ± SEM, n=4.

**Supplementary Figure 5. The 17β-estradiol (E2) treatment does not alter the proportion of follicular helper T (TFH) cells, germinal center (GC) B cells, and T follicular regulatory (TFR) cells in immunized CD4-ERα knockout (KO) female mice.**

E2 (200 µg) was injected daily to wild-type (WT) and CD4-ERα knockout (KO) female mice from 3 days prior of the NP-OVA immunization. After immunization, E2 was continuously injected daily for 7 days. The inguinal lymph nodes (ILNs) were analyzed by flow cytometry. **a** Flow cytometric analysis of CD4^+^CD44^+^CXCR5^+^Bcl-6^+^ TFH and B220^+^CD95^+^GL-7^+^ GC B cells in the ILN from 8-week-old NP-OVA-immunized CD4-ERα KO female mice with or without E2 treatment. Scattered graph shows the representative data. **b** Flow cytometric analysis of TFR cells in the ILN from 8-weeks-old NP-OVA-immunized CD4-ERα KO female mice with or without E2 treatment. Bar graph shows the representative data. Values represent the mean ± SEM, n=4.

**Supplementary Figure 6. ERα-deficiency in T cells does not alter the proportion of regulatory T cells in 1-year-old CD4-ERα knockout (KO) mice.**

**a, b** Lymphocytes from the spleen, mesenteric lymph nodes (MLN), and inguinal lymph nodes (ILN) were isolated from 1-year-old wild-type (WT) and CD4-ERα KO mice, and then stained with anti-CD4-PerCP-cy5.5 for 15 min at 4°C. The cells were fixed and permeabilized by the eBio Foxp3 fixation kit, and then immunostained by an anti-Foxp3-APC antibody for flow cytometric analysis. Values represent the mean ± SEM, n=5.

**Supplementary Figure 7. ERα would binds to ERE-like region on the front of Bcl-6 promoter**

Bcl-6 promoter sequence was identified based on UCSC Genome browser (<https://genome.ucsc.edu/>), and ERE-like region on the front of bcl-6 promoter (-585 ~ -574, -1229 ~ -1217) was identified.

**Supplementary Figure 8. Serum IFNγ level was increased in 1-year-old CD4-ERα knockout (KO) mice.**

The sera from 1-year-old wild-type (WT) and CD4-ERα KO male and female mice were collected by eye bleeding, and IFNγ and IL-13 cytokine levels were measured by ELISA following by manufacturer’s protocol. Values represent the mean ± SEM, n=5.

**Supplementary Figure 9. ERα-deficiency in T cells differs for Th1/Th2 differentiation.**

Magnetic-activated cell sorting (MACS)-sorted naïve CD4 T cells from wild-type (WT) and CD4-ERα knockout (KO) mice were differentiated into Th1, 2, 17, and Treg cells at day 3 (Th1, 17, and Treg) and day 6 (Th2). Differentiated T cells were re-stimulated by PMA/ionomycin with a protein transport inhibitor (BFA, Monensin), and intracellular cytokine production was analyzed by flow cytometry. Values represent the mean ± SEM, n=5, *P<0.05.

**Tables with titles and legends**

**Supplementary Table 1. Primer pairs for real-time PCR.**
