# Supplementary material for: Estrogen receptor α in T cells suppresses follicular helper T cell responses and prevents autoimmunity
Source: Exp Mol Med. 2019 Apr 15;51(4):41. doi: 10.1038/s12276-019-0237-z (PMC6465332; doi:10.1038/s12276-019-0237-z)
Supplement: Supplementary file 2 — Supplementary Table 1 [file 12276_2019_237_MOESM2_ESM.pdf]

Primer pair		Sequence (5' - 3')
Bcl-6	Forward	CCGGCACGCTAGTGATGTT
	Reverse	TGTCTTATGGGCTCTAAACTGCT
IL-21	Forward	CGCCTCCTGATTAGACTTCG
	Reverse	TGGGTGTCCTTTTCTCATACG
ERα	Forward	TCCAGCAGTAACGAGAAAGGA
	Reverse	AGCCAGAGGCATAGTCATTGC
ERβ	Forward	CTGTGCCTCTTCTCSCSSGGA
	Reverse	TGCTCCAAGGGTAGGATGGAC
β-Actin	Forward	TGTCCCTGTATGCCTCTGGT
	Reverse	CACGCACGATTTCCCTCTC
