# Supplementary figures and images for: Estrogen receptor α in T cells suppresses follicular helper T cell responses and prevents autoimmunity
Source: Exp Mol Med. 2019 Apr 15;51(4):41. doi: 10.1038/s12276-019-0237-z (PMC6465332; doi:10.1038/s12276-019-0237-z)

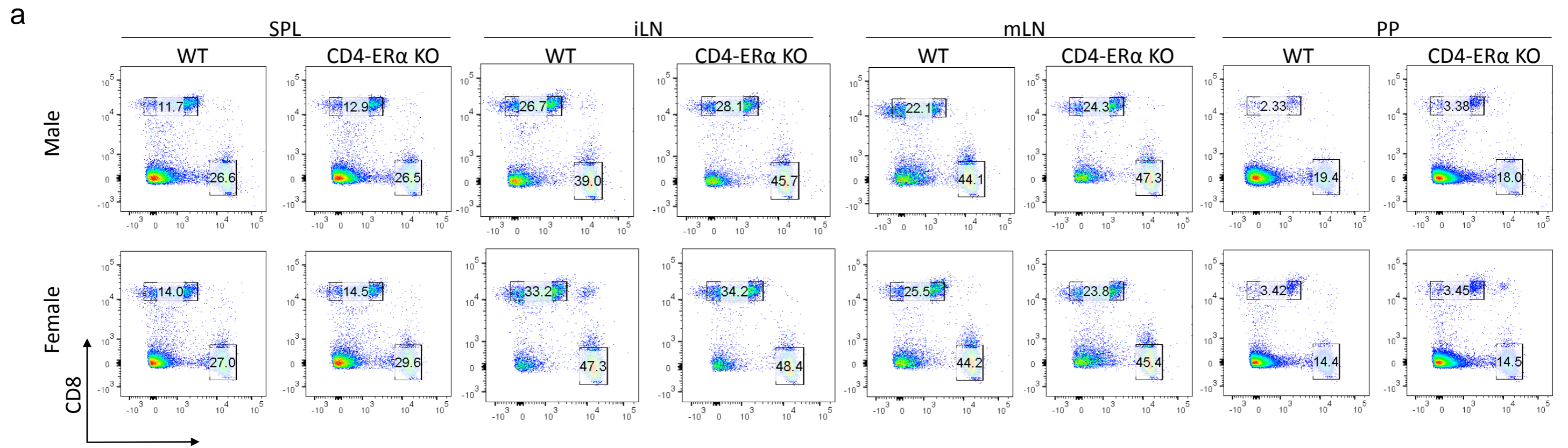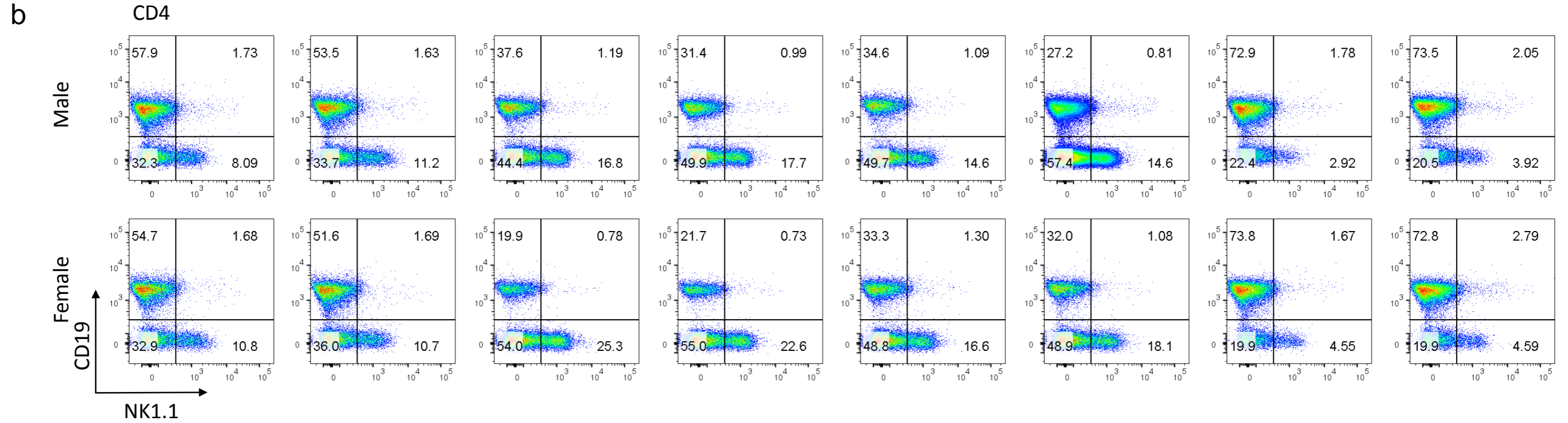

Supplement: Supplementary file 3 — Supplementary Figure 1 [file 12276_2019_237_MOESM3_ESM.pdf]

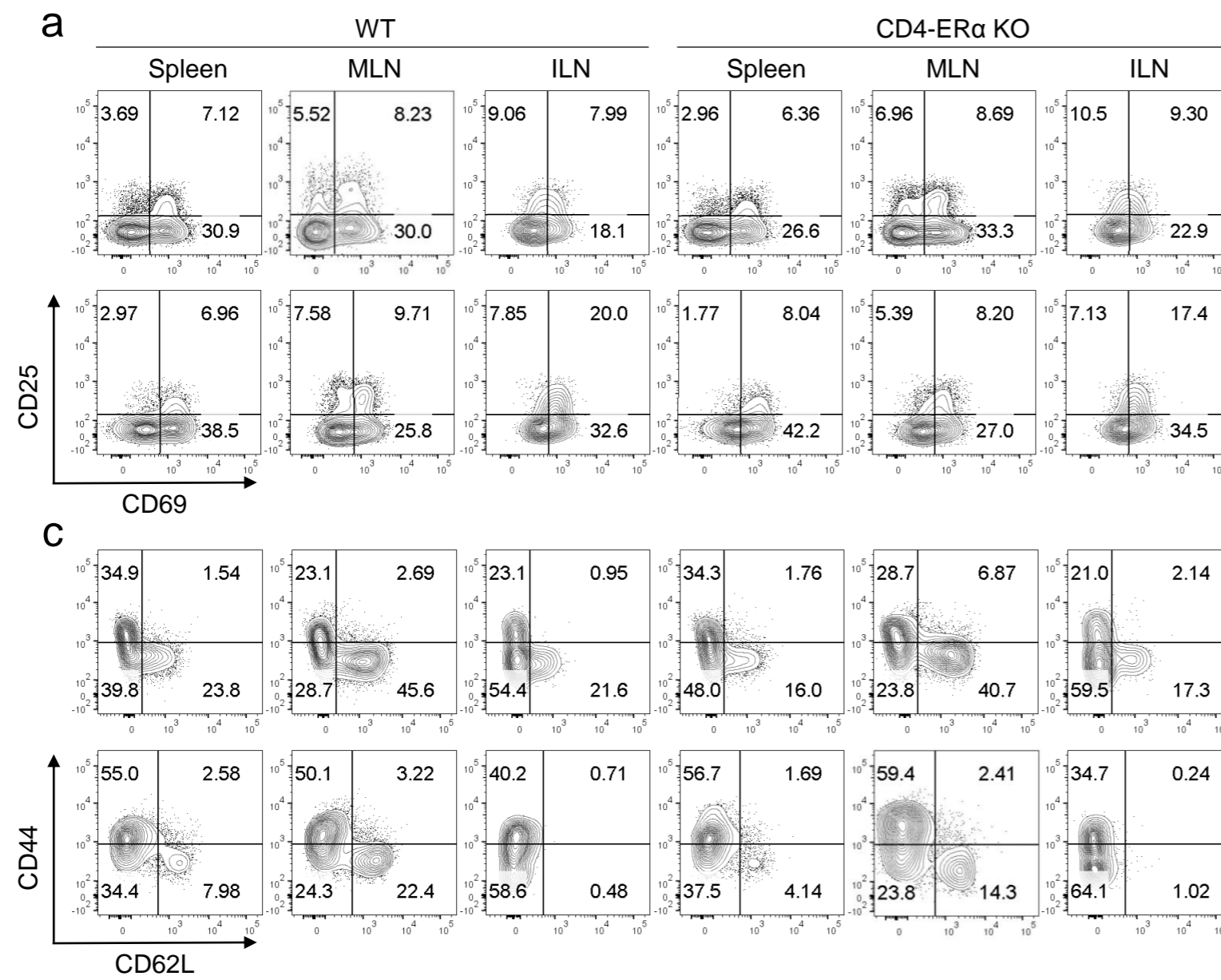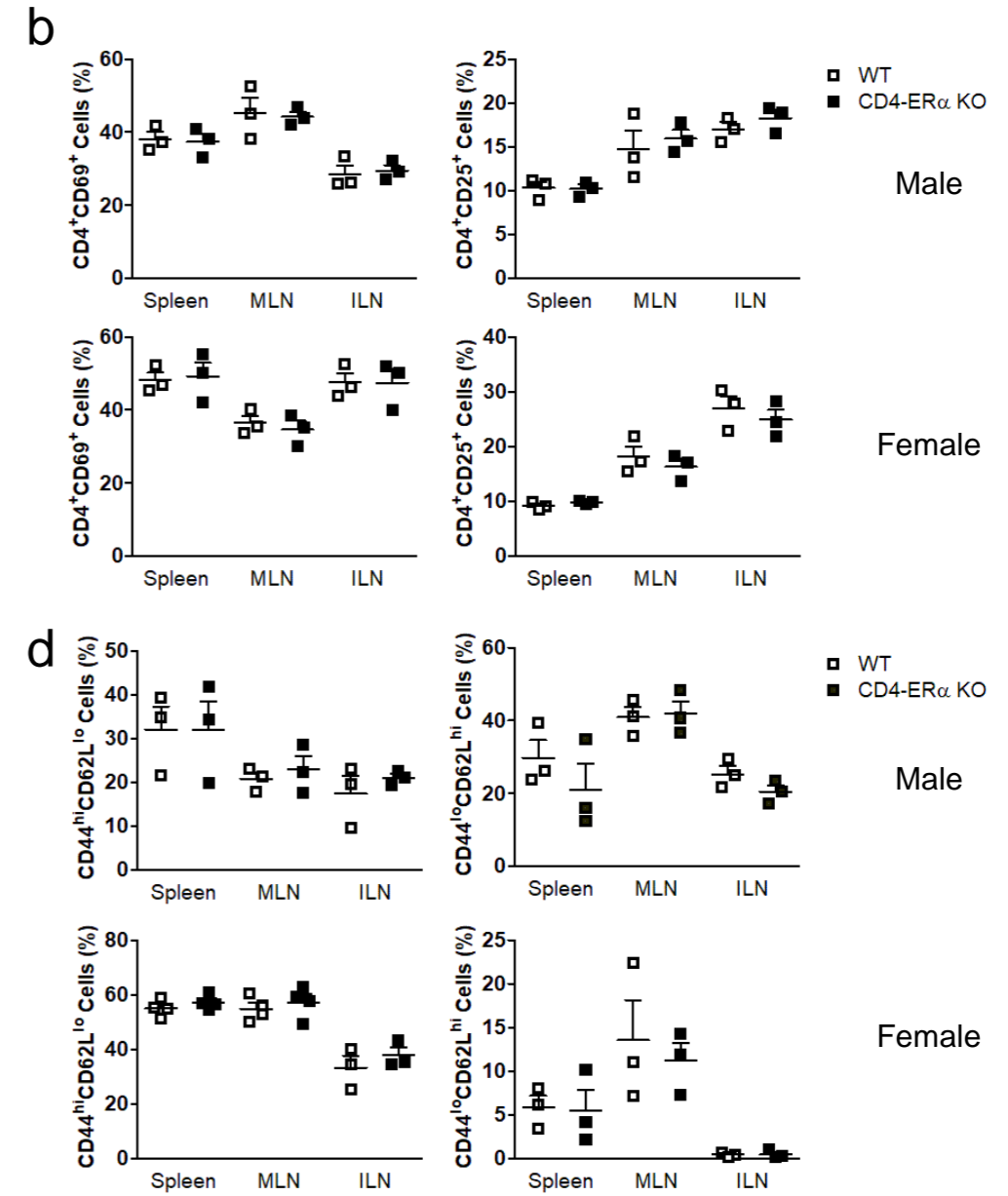

Supplement: Supplementary file 4 — Supplementary Figure 2 [file 12276_2019_237_MOESM4_ESM.pdf]

a

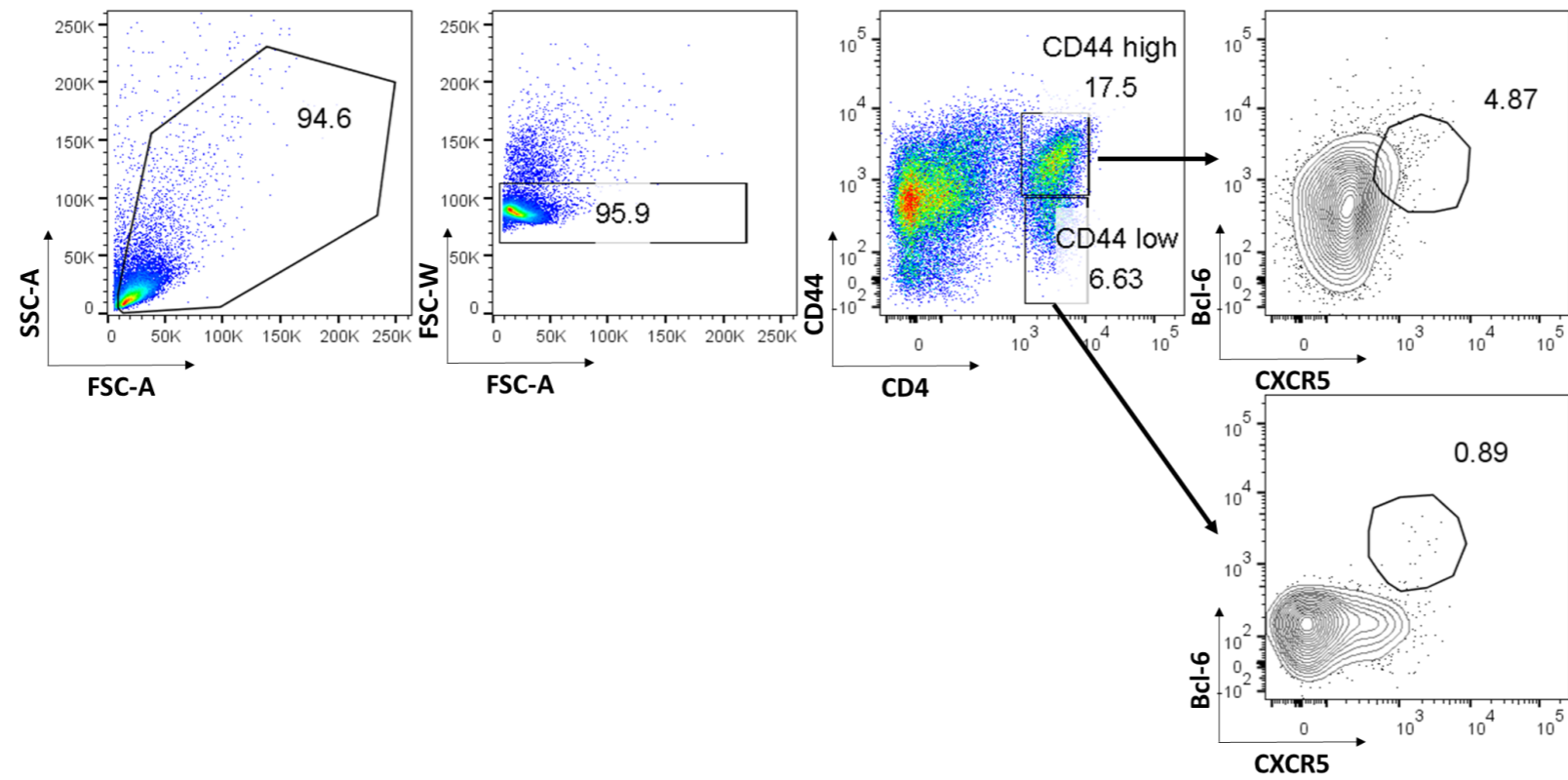

b

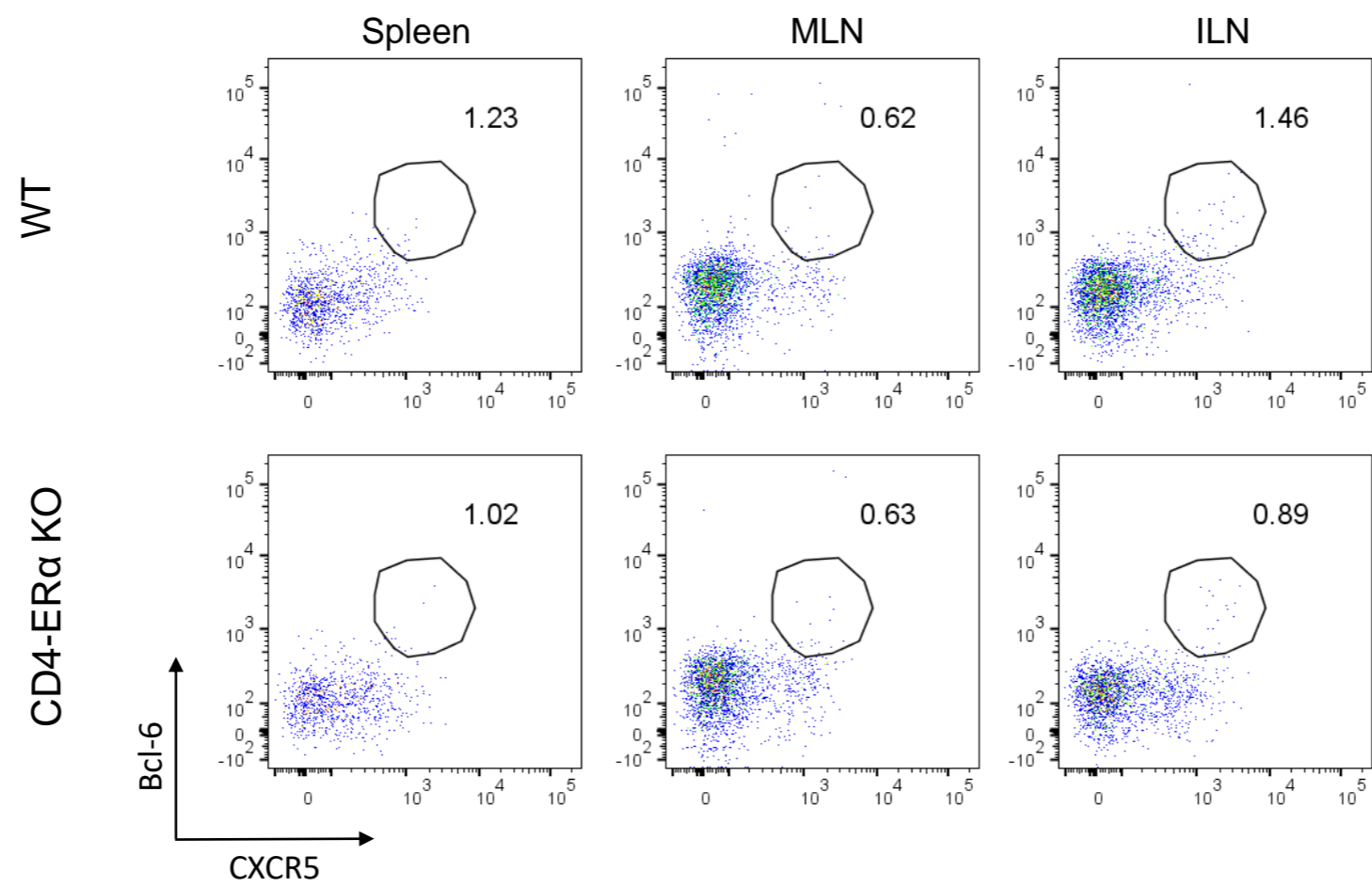

Supplement: Supplementary file 5 — Supplementary Figure 3 [file 12276_2019_237_MOESM5_ESM.pdf]

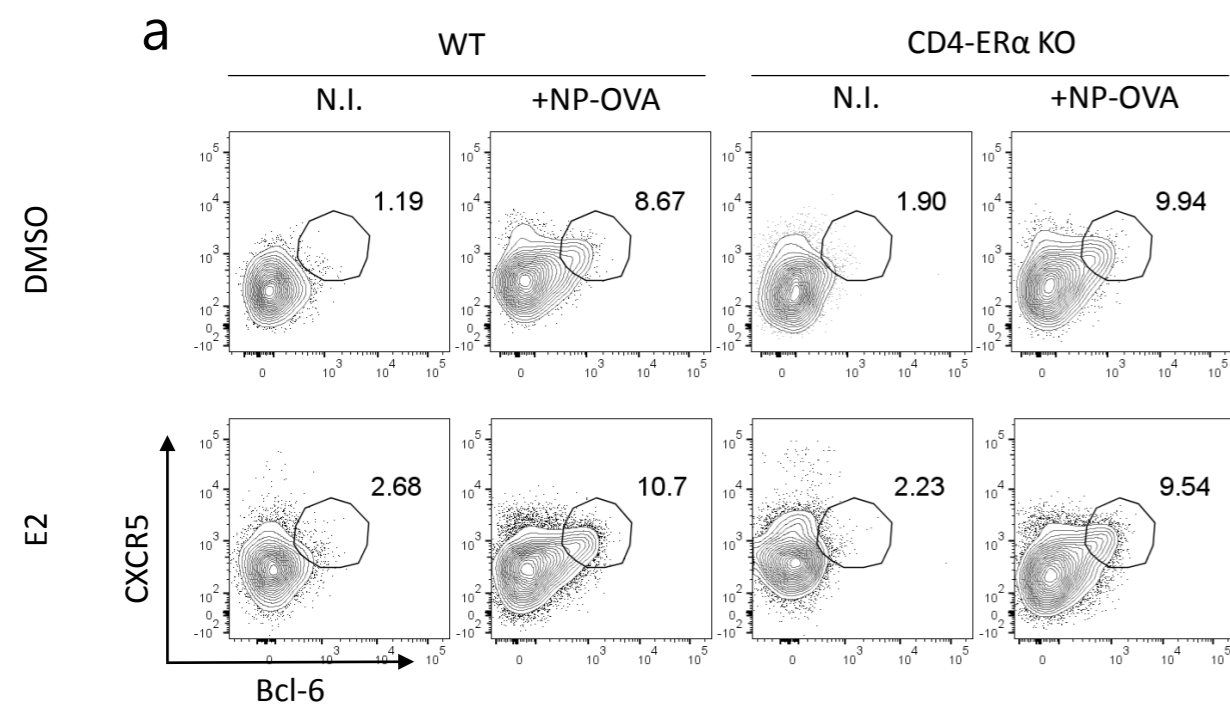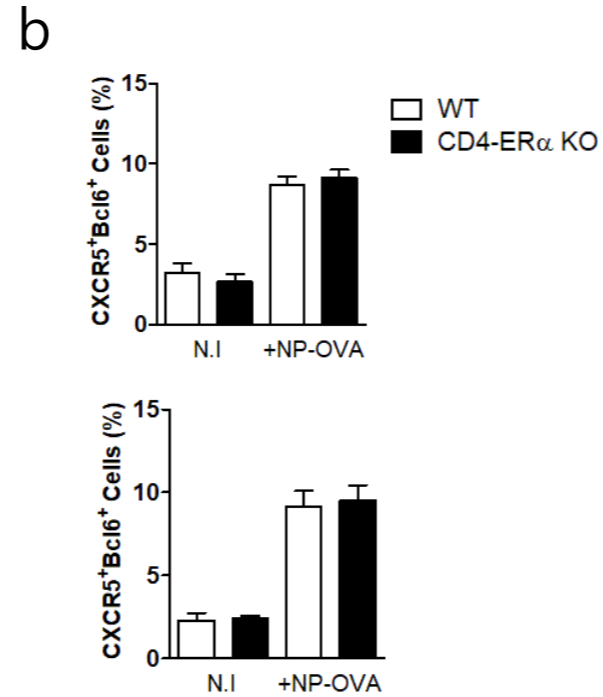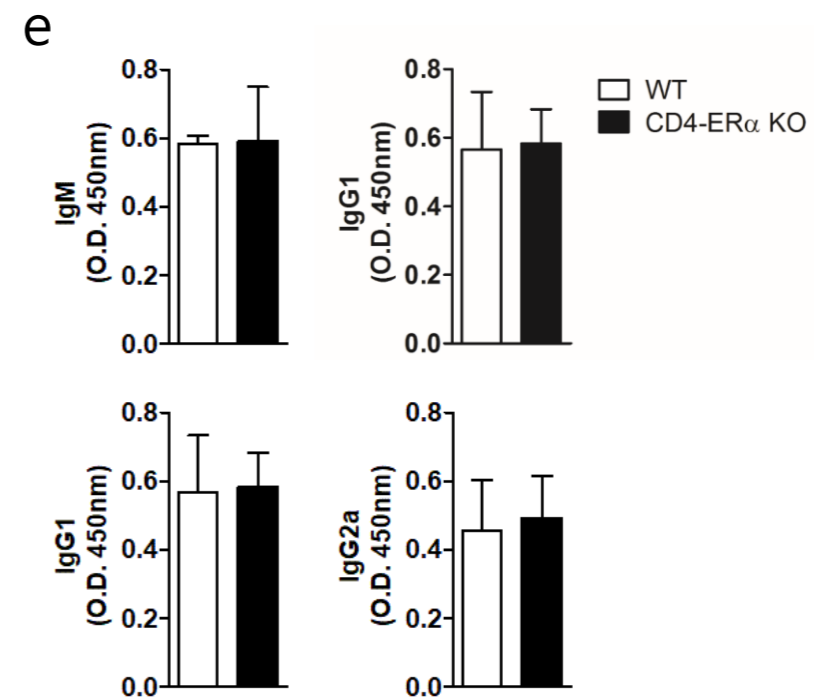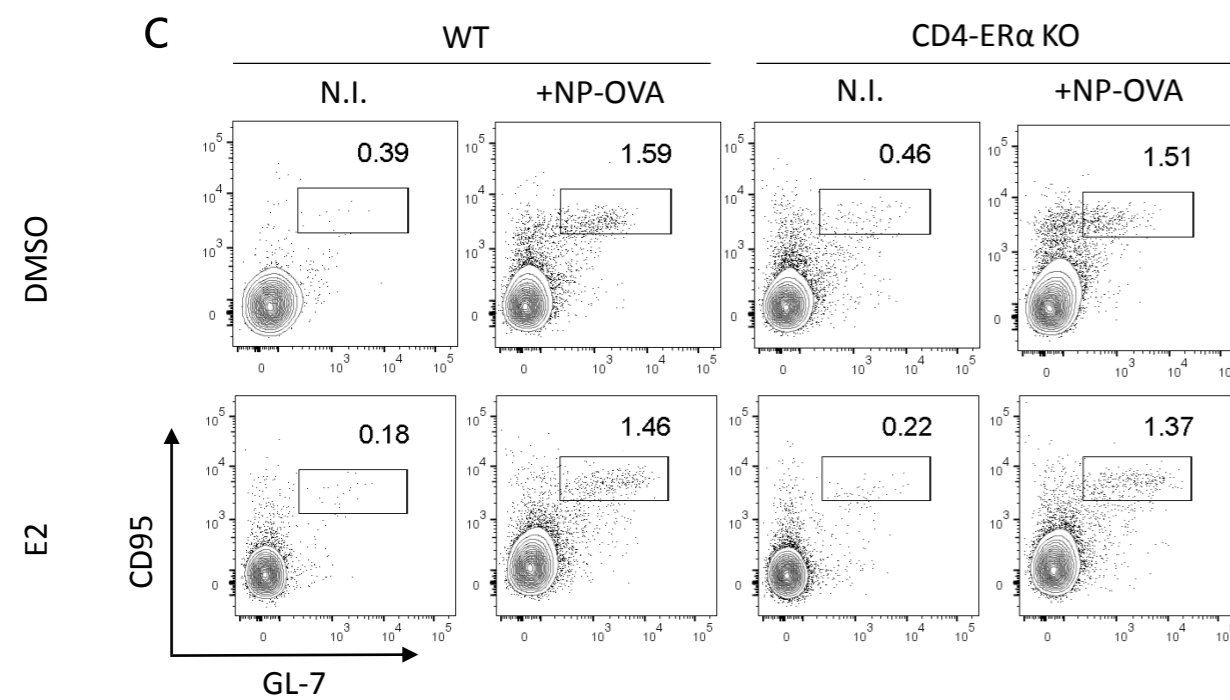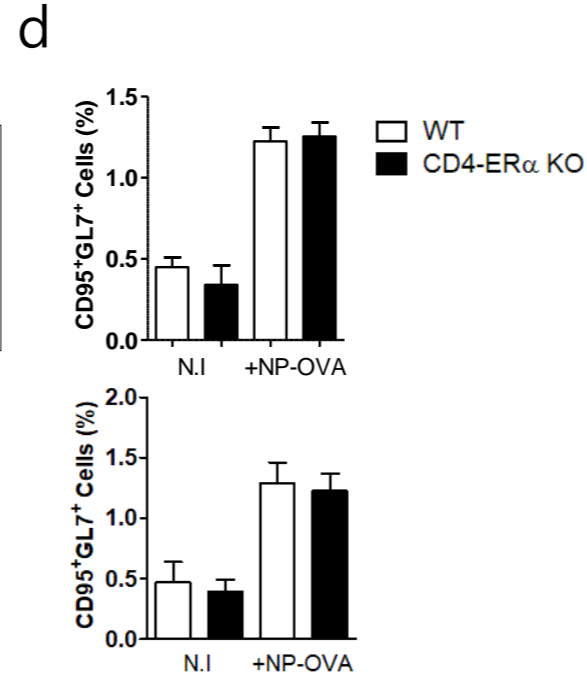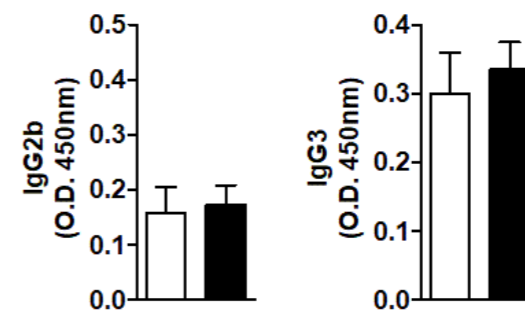

Supplement: Supplementary file 6 — Supplementary Figure 4 [file 12276_2019_237_MOESM6_ESM.pdf]

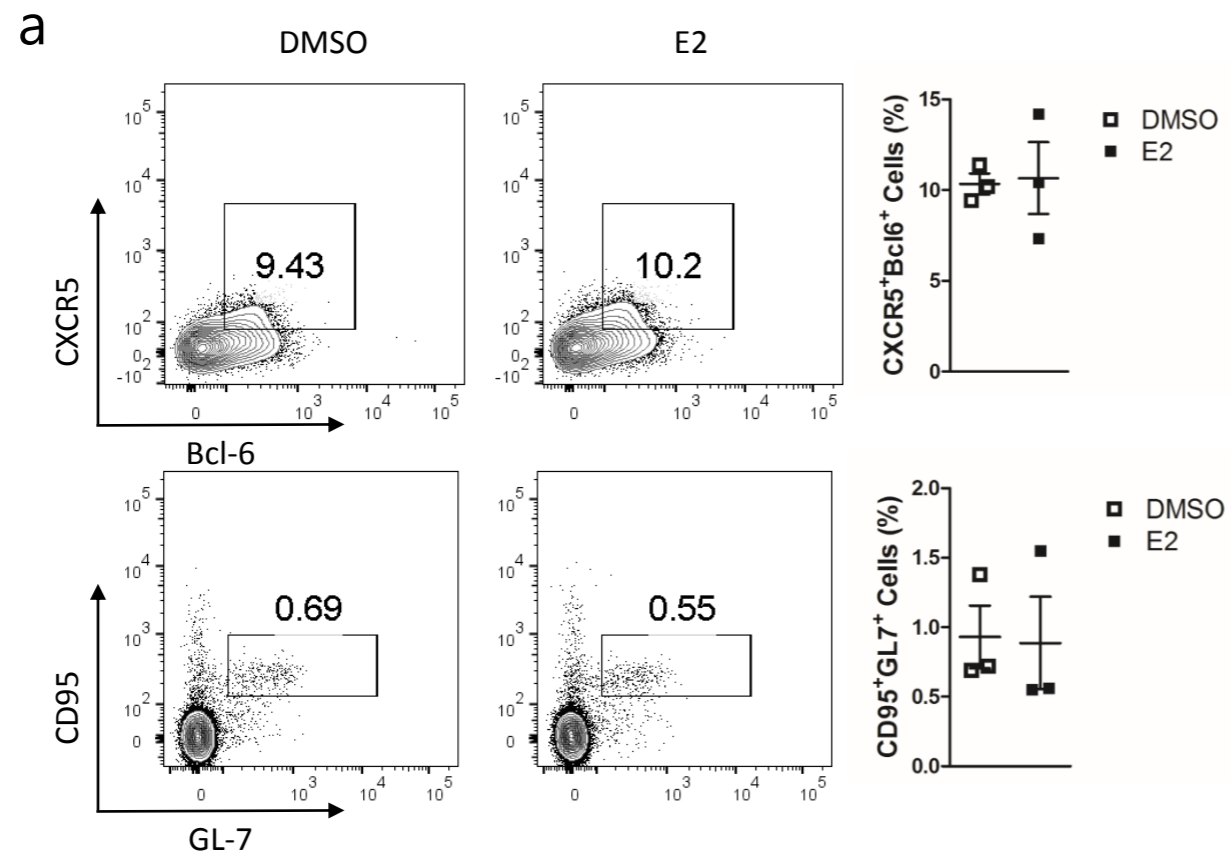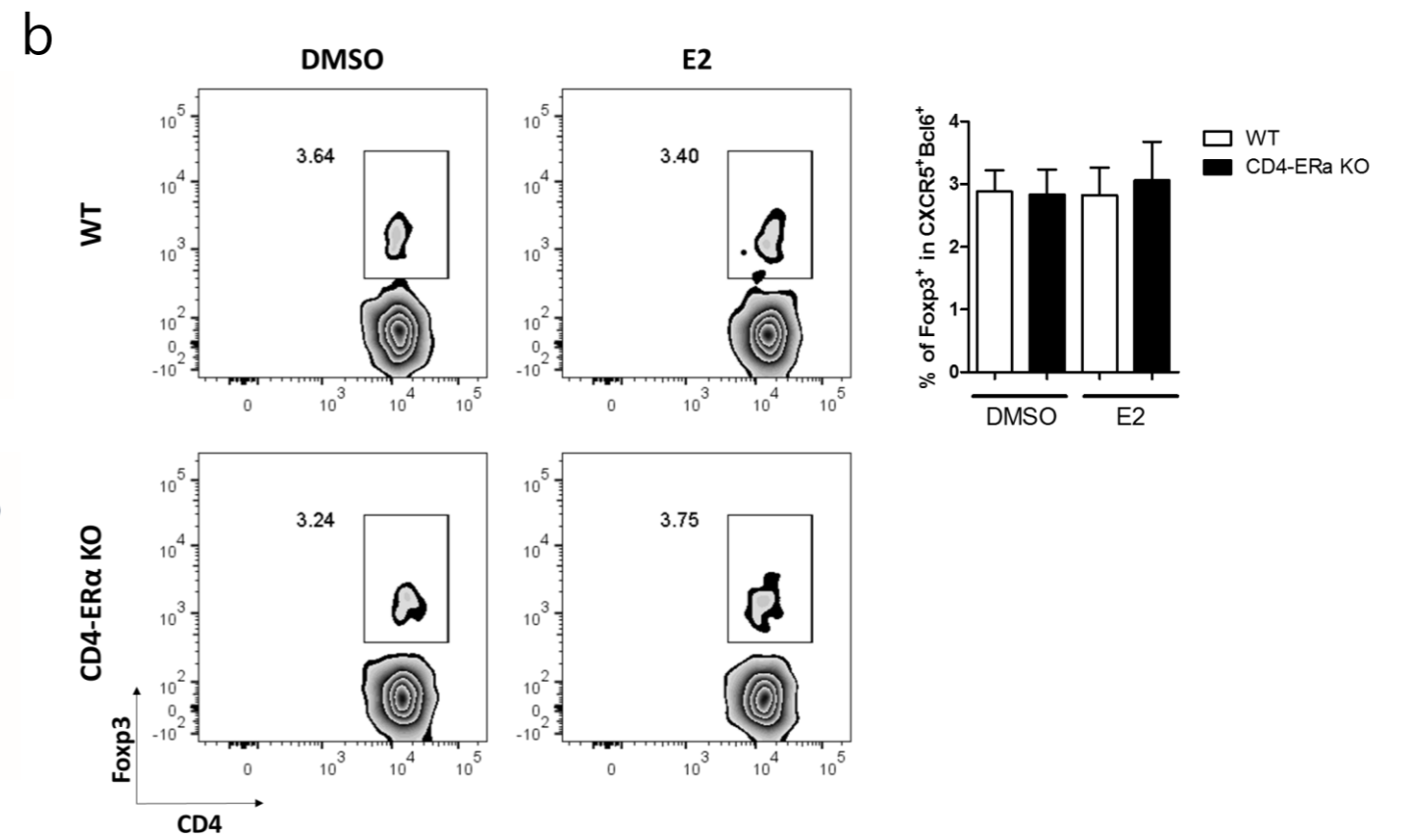

Supplement: Supplementary file 7 — Supplementary Figure 5 [file 12276_2019_237_MOESM7_ESM.pdf]

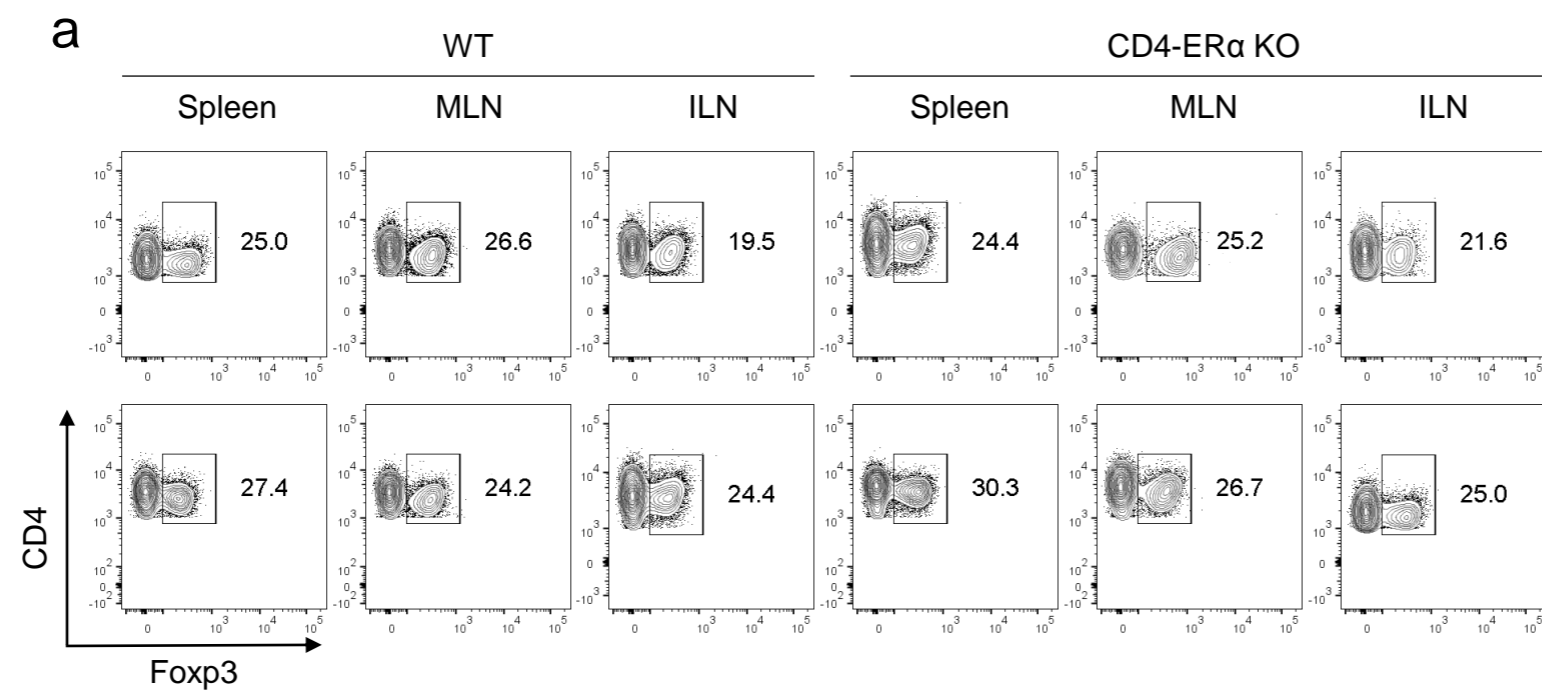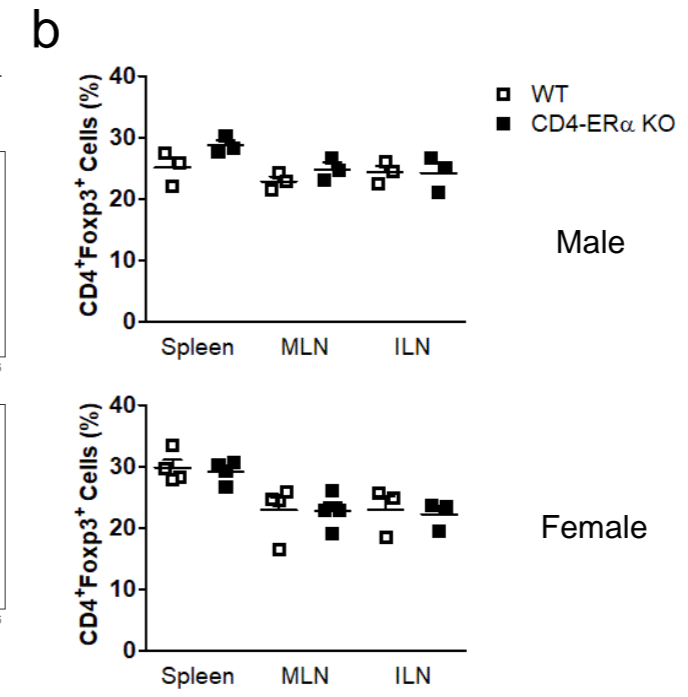

Supplement: Supplementary file 8 — Supplementary Figure 6 [file 12276_2019_237_MOESM8_ESM.pdf]

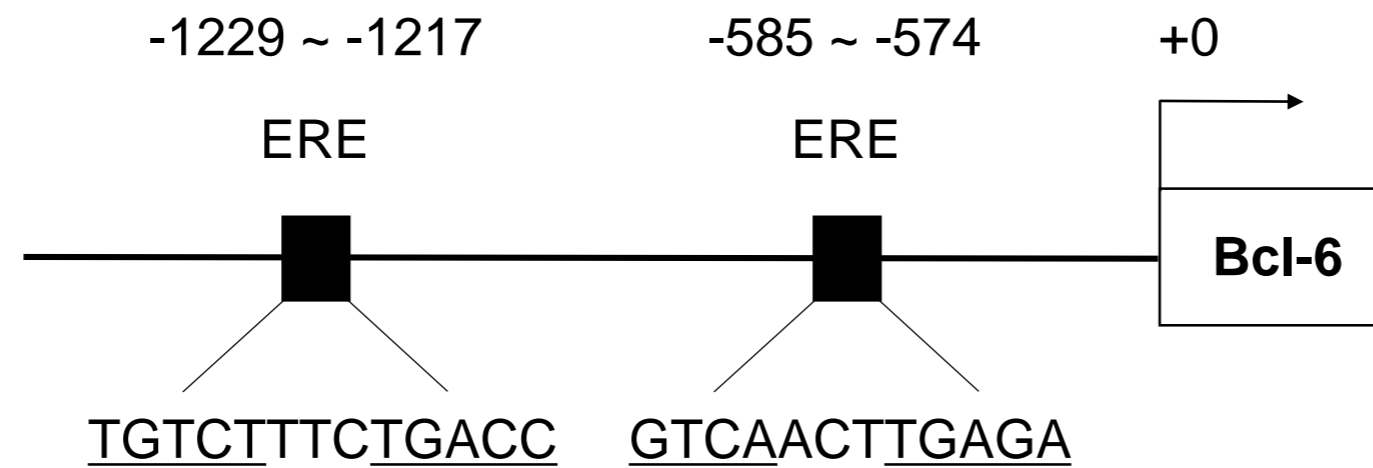

Supplement: Supplementary file 9 — Supplementary Figure 7 [file 12276_2019_237_MOESM9_ESM.pdf]

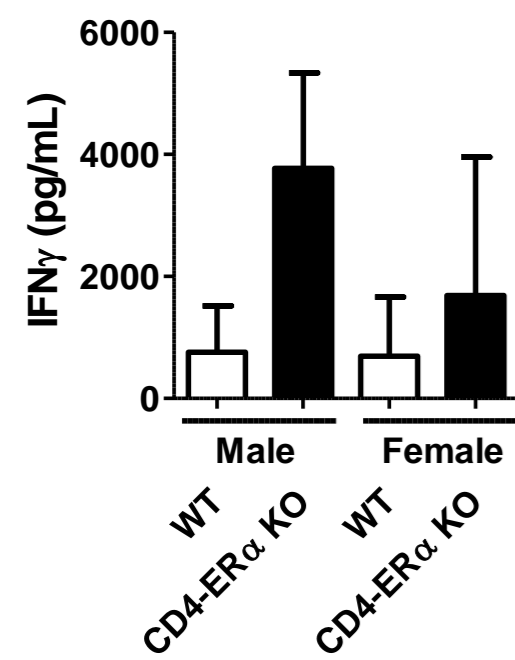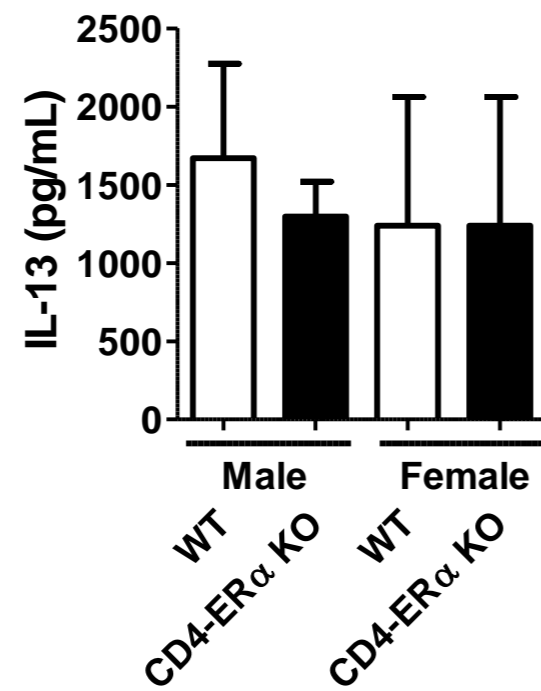

Supplement: Supplementary file 10 — Supplementary Figure 8 [file 12276_2019_237_MOESM10_ESM.pdf]

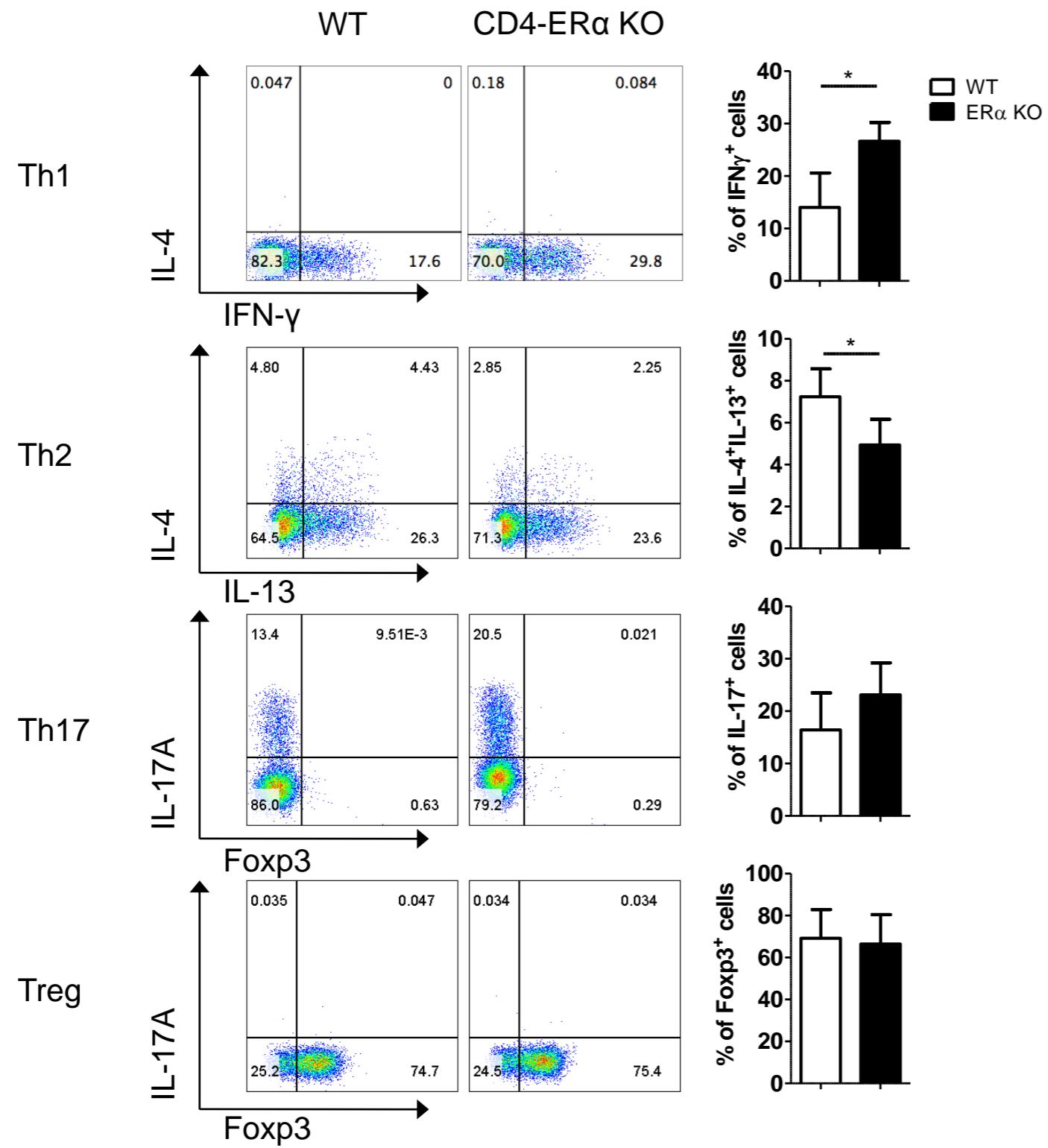

Supplement: Supplementary file 11 — Supplementary Figure 9 [file 12276_2019_237_MOESM11_ESM.pdf]
